# Predicted and inducible prophages display contrasting virulence gene profiles within the prophage–SaPI mobilome of *Staphylococcus aureus*

**DOI:** 10.1128/msphere.00103-26

**Published:** 2026-03-31

**Authors:** Alan Aguayo-González, Irma Martínez-Flores, Patricia Bustos, Rosa I. Santamaría, Roberto Cabrera-Contreras, Areli Martínez-Gamboa, Rodrigo Ibarra-Chávez, Víctor González

**Affiliations:** 1 Centro de Ciencias Genómicas, Universidad Nacional Autónoma de México 61740 , Cuernavaca, Morelos, México; 2 Laboratorio de Patogenicidad Bacteriana, Departamento de Salud Pública, Facultad de Medicina, Universidad Nacional Autónoma de México, Ciudad de México, México; 3 Instituto Nacional de Ciencias Médicas y Nutrición Salvador Zubirán, Ciudad de México, México; 4 Section of Microbiology, Department of Biology, University of Copenhagen 4321, Copenhagen, Denmark; 5 Center for Evolutionary Hologenomics, Globe Institute, University of Copenhagen 4321, Copenhagen, Denmark; University of Galway, Galway, Ireland

**Keywords:** *Staphylococcus*, prophages, phage-inducible chromosomal islands, SaPIs

## Abstract

Prophages play a significant role in bacterial evolution by shaping genomic diversity, virulence, and host adaptation. This study investigated the prophage composition of 109 clinical *Staphylococcus aureus* isolates obtained from four tertiary care hospitals in Mexico City and compared these results with data from 993 global genomes. Prophages were present in 97% of local isolates. Consistently, analysis of the global genome collection revealed a 99% prevalence, supporting the near ubiquity of prophages in *S. aureus*. Analysis identified 216 genomic regions corresponding to the predicted prophages within the Mexican *S. aureus* isolates. A substantial fraction (19%) of the predicted prophages was identified as phage-inducible chromosomal islands (PICIs), such as SaPI1, SaPI2, and SaPIpt1028-like elements. These PICIs encoded anti-phage defense systems (63%) and virulence genes (27%). Experimental treatment with mitomycin C induced 17 temperate phages, of which 12 demonstrated functional activity and the ability to undergo lysogenic-lytic switching and reinfection. No virulence or antibiotic resistance genes were identified in these temperate phages. Conversely, several uninduced prophages coincided with the virulence determinants. These findings highlight the complexity of the *S. aureus* mobilome, characterized by distinct functional profiles and heterogeneous mobilization capabilities, which may influence the dissemination of virulence factors.

**IMPORTANCE:**

*Staphylococcus aureus* is a significant hospital-associated pathogen whose evolutionary processes are shaped by mobile genetic elements, including prophages and phage-inducible chromosomal islands (PICIs). While computational analyses suggest that nearly all *S. aureus* genomes contain prophages, our findings indicate that only a subset is inducible following mitomycin C treatment. These temperate phages do not possess virulence genes; however, other predicted prophages are associated with virulence factors. Additionally, we identified numerous predicted prophages as PICIs, which harbored anti-phage defense mechanisms and toxins. This study highlights the intricate mobilome of *S. aureus* and the various strategies that contribute to its horizontal gene transfer and pathogenic evolution.

## INTRODUCTION

*Staphylococcus aureus* is a globally significant opportunistic pathogen responsible for a broad spectrum of infections, ranging from superficial skin and soft tissue infections to severe and life-threatening diseases, such as pneumonia, endocarditis, and sepsis. A defining feature of *S. aureus* is its remarkable genomic plasticity, which is largely driven by the acquisition and diversification of mobile genetic elements (MGEs). These include bacteriophages (phages), phage-inducible chromosomal islands (PICIs), staphylococcal chromosomal cassettes (*SCCmec*), transposons, insertion sequences, conjugative plasmids, and genomic islands, all of which contribute to virulence, antimicrobial resistance, and adaptation to diverse ecological niches, particularly in hospital environments (1, 2).

Among these MGEs, temperate phages play a central role in shaping the *S. aureus* population structure by mediating the horizontal gene transfer of virulence-associated traits (3). Although only a limited number of *S. aureus* phages have been experimentally characterized, several well-studied examples, such as ϕSa3, ϕ11, ϕ80, and ϕMu50, are known to encode key virulence determinants (4–6). These include immune evasion factors (e.g., *sak*, *scn*, and *chp*), toxins such as Panton–Valentine leukocidin (*lukF-PV* and *lukS-PV*), and superantigens (*sea*, *sep*, and *sec*), which are frequently associated with epidemic lineages, including clonal complexes CC5, CC8, and CC30 (7–9). In contrast, the prophage repertoire and functional contributions of phages associated with methicillin-susceptible *S. aureus* (MSSA) lineages remain comparatively understudied.

Chromosomally integrated prophages are nearly ubiquitous in *S. aureus*, with most genomes harboring one to four prophage elements (10). While prophages rarely encode antibiotic resistance genes, bioinformatic analyses have predicted that many carry virulence-associated functions (11). However, genome-based predictions frequently identify both intact and fragmented prophage regions, making it difficult to determine which elements remain functionally active, that is, those capable of excision, replication, and production of infectious viral particles through induction of the lytic cycle.

Prophage functionality is commonly assessed experimentally using induction assays, typically using mitomycin C (MMC), a DNA-damaging agent that activates the bacterial SOS response and promotes repressor inactivation and prophage excision. MMC is a natural antibiotic widely employed to identify inducible prophages in *S. aureus* isolates of human and veterinary origin (12, 13). Additional stressors with greater ecological or clinical relevance, including fluoroquinolone antibiotics, oxidative stress–associated metabolites such as pyocyanin produced by *Pseudomonas aeruginosa*, and quorum-sensing signals mediated by peptide autoinducers, have also been reported to influence prophage induction in *S. aureus* and related bacteria (14–16). To date, inducible *S. aureus* phages characterized under these conditions largely belong to the class *Caudoviricetes* and generally lack known virulence or antibiotic resistance determinants.

Beyond their direct role as vectors, phages also facilitate the mobilization of other MGEs, particularly PICIs, which are best exemplified by pathogenicity islands (SaPIs) in *S. aureus*. SaPIs encode potent virulence factors and exploit helper phages for excision, replication, and encapsidation, frequently redirecting phage capsid assembly to favor SaPI DNA packaging (2, 17). This phage–SaPI interaction enables highly efficient horizontal gene transfer and is considered a major driver of virulence dissemination within *S. aureus* population (18–20).

Hospital environments constitute ecological settings in which *S. aureus* is exposed to strong and recurrent selective pressures, including antibiotic treatment and host immune responses. Under these conditions, prophages and SaPIs may play a critical role in bacterial adaptation by facilitating the horizontal transfer of virulence-associated genes. However, it remains unclear which prophage elements circulating in clinical *S. aureus* populations are functionally inducible and capable of mediating gene transfer under clinically relevant stress conditions (21).

In this study, we combined comparative genomics and experimental induction assays to investigate the diversity, functionality, and mobilization potential of prophages and SaPIs in *S. aureus* clinical isolates recovered from tertiary care hospitals in Mexico City, Mexico. We showed that only a subset of predicted prophages is functionally active and capable of producing infectious particles and establishing lysogeny following MMC induction. Although these inducible phages lacked known virulence or antibiotic resistance genes, we identified a substantial contribution of SaPIs embedded within prophage-rich genomic regions, supporting the continued role of phage–SaPI interactions as a key mechanism driving virulence gene dissemination in clinical *S. aureus* populations.

## RESULTS

### Global and local prophage diversity in *S. aureus*

We analyzed a global collection of 993 complete *S. aureus* genomes from GenBank to assess prophage prevalence and diversity. Using VIBRANT v1.2.1 (22), prophage regions were detected in 99% of the genomes, with most strains harboring one to four elements, confirming the near ubiquity of prophages in this species (see Table S1 at https://github.com/aguayo-alan/Aguayo-Gonzalez_et_al_mSphere_2026). Of the 3,279 predicted regions, 1,972 high- and medium-quality prophages were retained after filtering the low-quality predictions. This data set of prophage genomes exhibited size and structural characteristics comparable to those of experimentally validated reference *S. aureus* phage genomes available in the NCBI Viral Genome Database (see Fig. S1A at https://github.com/aguayo-alan/Aguayo-Gonzalez_et_al_mSphere_2026).

However, phage-related mobile elements, such as SaPIs, may confound the identification of prophages. Thus, to investigate the potential occurrence of SaPIS within the VIBRANT prophage predictions, a BlastN search for SaPI integrases was performed (see Table S5 at https://github.com/aguayo-alan/Aguayo-Gonzalez_et_al_mSphere_2026; also see Materials and Methods). This analysis revealed 222 SaPI integrases, indicating a potential limitation in the accuracy of the VIBRANT prophage prediction tool used.

Next, we examined prophage diversity at a local scale by analyzing 109 *S. aureus* clinical isolates recovered from patients treated in tertiary care hospitals across Mexico City (see Table S2 at https://github.com/aguayo-alan/Aguayo-Gonzalez_et_al_mSphere_2026). Prophage regions were detected in 97% (106/109) of genomes. The three genomes lacking detectable prophages (INPER770, INPER772, and INPER775) corresponded to MSSA isolates belonging to clonal complex 5 (CC5), a lineage that otherwise harbored prophages in multiple strains, suggesting a strain-specific rather than a lineage-wide absence.

In total, 519 prophage regions were identified in the local data set, of which 216 high-quality and medium-quality prophages exhibited genome size distributions comparable to those observed in the global collection and experimentally characterized *S. aureus* phages (see Fig. S1B at https://github.com/aguayo-alan/Aguayo-Gonzalez_et_al_mSphere_2026). In this curated set, 41 elements were identified as SaPI integrases.

### Prophages related to MRSA isolates

**Fig 1:**
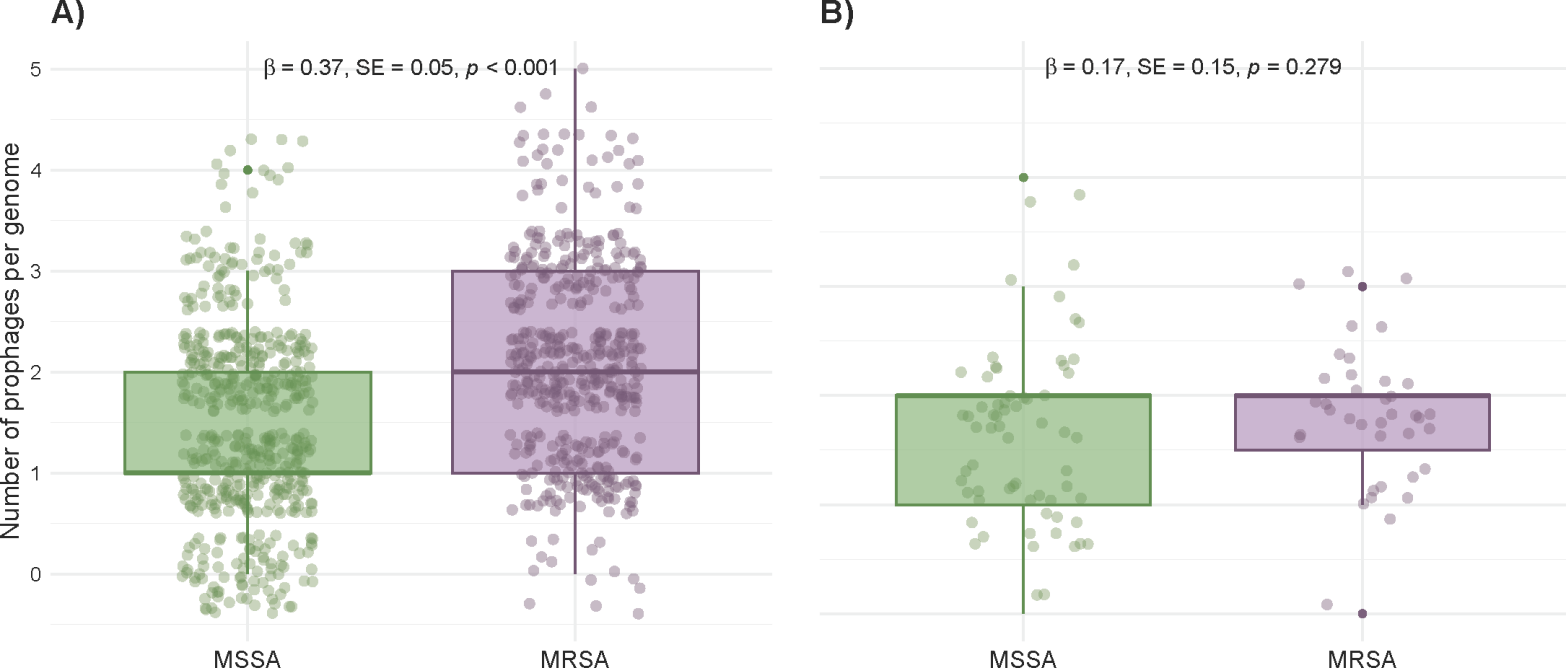
Distribution of prophage content in methicillin-resistant and -susceptible *S. aureus* isolates. Box plots show the number of prophages per genome in MRSA (purple) and MSSA (green) strains from international (**A**) and local collections (**B**). High- and medium-quality VIBRANT predictions were included (see Materials and Methods). Statistical comparisons were performed using generalized linear mixed-effects models, accounting for clonal complex structure; model estimates and *P*-values are annotated in the figure.

To evaluate whether prophage content differed between methicillin-resistant (*S. aureus*, MRSA) and methicillin-susceptible (*S. aureus*, MSSA) strains of the global collection, we used two approaches: a simple distribution of the predicted prophages in MRSA and MSSA genomes, which indicated that the former harbored more predicted prophages (Wilcoxon *P* = 3.6 × 10⁻¹⁷) (see Fig. S2 at https://github.com/aguayo-alan/Aguayo-Gonzalez_et_al_mSphere_2026). However, highly similar strains, likely the same clones, may introduce a bias in counting prophages in similar genomic backgrounds. To correct for this possibility, we used a statistical model (generalized linear mixed-effects model [GLMM]) to correct for clonal complexes (see Materials and Methods). This analysis revealed a significant association between methicillin resistance and prophage abundance: MRSA genomes harbored a higher number of prophages than MSSA genomes (β = 0.3667 ± 0.0538 SE, *z* = 6.82, *P* < 0.001), corresponding to a 1.44-fold increase and estimated marginal means of 2.05 prophages per MRSA genome versus 1.42 per MSSA genome (Fig. 1A).

The observed differences imply a possible link between prophage abundance and methicillin resistance, but not necessarily prophage diversity. To address this possibility, we evaluated prophage diversity by clustering high- and medium-quality sequences using vConTACT2 (23, 24), which groups viral genomes based on shared protein content and similarity. Prophage sequences formed 124 viral clusters (VCs): 35 contained only MRSA prophages, 43 contained only MSSA, and 46 contained both. While some lineages appear more common in MRSA or MSSA strains, these trends may reflect clonal population structure, sampling bias, or lineage-specific phage exposure rather than an association with the resistance phenotype.

Using the same statistical framework applied to the global collection, we assessed the prophage abundance in local MRSA and MSSA isolates. Although MRSA genomes showed a slightly higher estimated mean number of prophages than MSSA genomes (1.82 vs 1.54; Fig. 1B), this difference was not statistically significant (β = 0.1655 ± .1528 SE; *z* = 1.08; *P* = 0.279).

Together, these results demonstrate that *S. aureus* genomes carry a diverse and largely conserved repertoire of prophages with variable abundance in global and local contexts. Importantly, the widespread presence of structurally complete prophages provides a basis for examining their functional potential.

### Prophage induction in clinical *S. aureus* isolates

**Fig 2:**
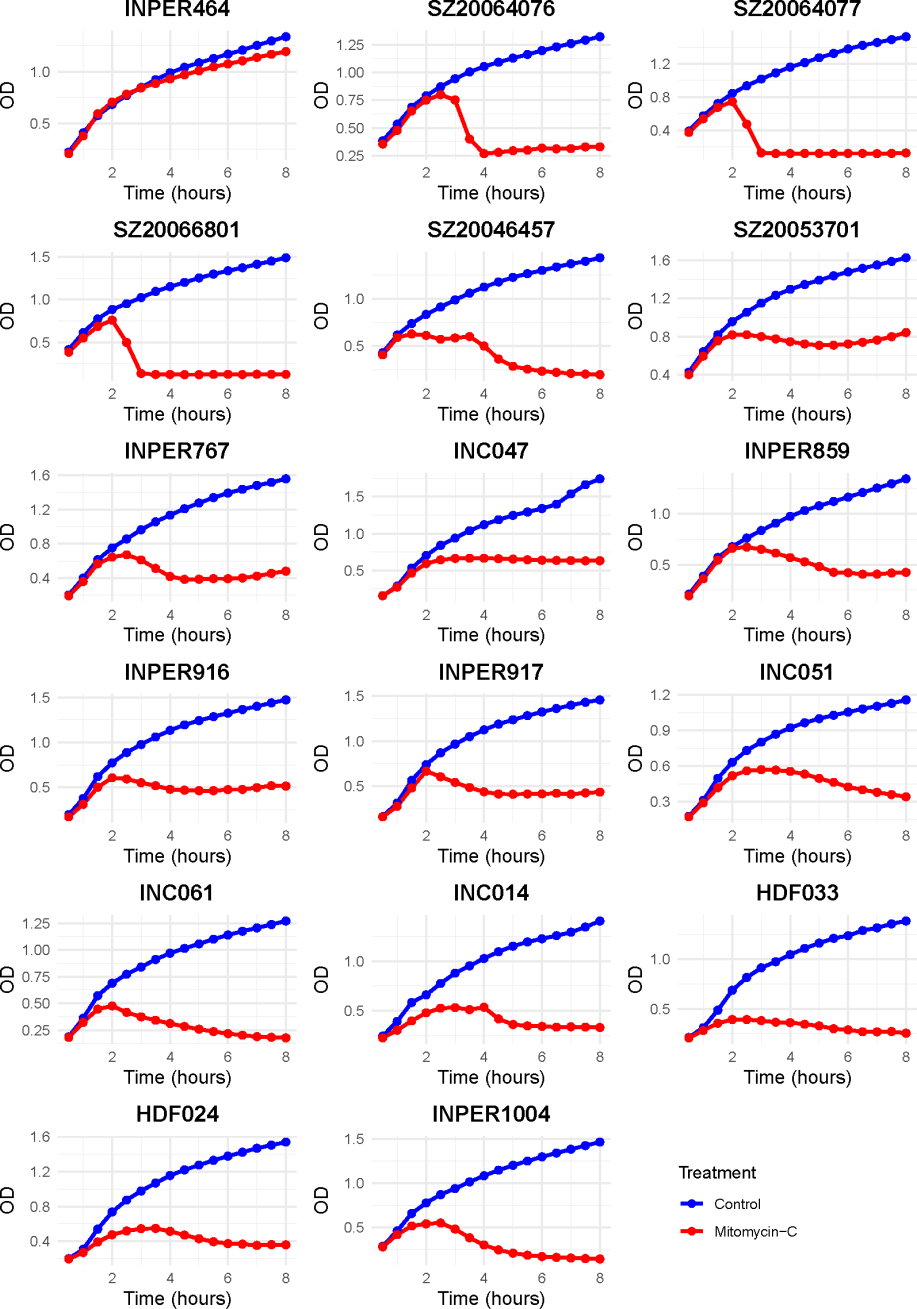
*S. aureus* growth kinetics with and without mitomycin C treatment.*S. aureus* growth in broth cultures was recorded by the change in optical density (OD560) over incubation time (8 h). The blue curves represent control growth (no treatment), whereas the red curves represent growth with MMC. The first graph (top left) shows the control strain (INPER464), which displayed no growth inhibition or prophage induction after MMC exposure. The remaining graphs depict the strains in which the 17 phages were successfully induced in this study.

Although bioinformatic tools can reliably predict the presence of multiple phages and phage-related elements in *S. aureus* genomes, these *in silico* approaches offer limited insights into the functional state of the predicted prophages, particularly their ability to excise and produce infectious phage particles upon induction (10, 25, 26). To address this limitation, we experimentally assessed the presence of inducible prophages in 109 clinical *S. aureus* isolates by treating bacterial cultures with MMC (0.5 μg/mL). Following treatment, 44% of the strains (48/109) exhibited a marked reduction in growth compared to the untreated controls (Fig. 2), consistent with potential prophage induction and associated with cellular stress.

Due to the absence of a susceptible *S. aureus* host strain for assessing the presence of infectious viral particles in MMC-induced cultures, we sought alternative strains capable of detecting these particles through double-layer spot assays. Notably, 58% of the lysates (28 out of 48) demonstrated measurable lytic activity against at least one of the 40 strains, as evidenced by the formation of clear or turbid plaques, indicating the release of infectious phages.

To recover individual phage particles or virions, the lysates were subjected to three rounds of plaque purification, resulting in the isolation of 17 distinct phages (see Fig. S3 at https://github.com/aguayo-alan/Aguayo-Gonzalez_et_al_mSphere_2026). Notably, 11 lysates did not produce plaques in the spot assay. This suggests that either no phage was present in the lysate or that there was no host-susceptible strain in the panel for these phages.

These results confirm that a substantial proportion of clinical *S. aureus* strains harbor prophages that are not only genetically identifiable but also functionally inducible, highlighting their potential roles in horizontal gene transfer and bacterial lysis under stress conditions.

### Lysogenic properties of the induced prophages

To assess the potential of the 17 purified phages to establish lysogeny in a new *S. aureus* host strain and subsequently revert to the lytic cycle, we generated lysogenic strains incorporating each of the 17 phages. Briefly, phage lysates were spotted onto lawns of selected susceptible *S. aureus* strains, and viable colonies emerging from the lysis zones were recovered and tested for phage resistance as an initial indicator of potential lysogen formation (see Materials and Methods). Four host strains (INPER775, ATCC 29213, SZ20054337, and INC047) were selected based on their susceptibility to the 17 phages and their host range profiles (see Fig. S4 at https://github.com/aguayo-alan/Aguayo-Gonzalez_et_al_mSphere_2026). INPER775 was included because of the absence of predicted prophage regions, whereas ATCC 29213 and SZ20054337 were selected after failing to yield inducible prophages after MMC treatment. Although INC047 harbored the MMC-inducible prophage phSaQ, it was selected because of its high susceptibility to phSaJ3. This phage was unable to infect and reproduce in any other host strain. Using these strains, we assembled a panel of 31 phage–host combinations (17 phages against one to two strains per phage, when applicable).

**Fig 3:**
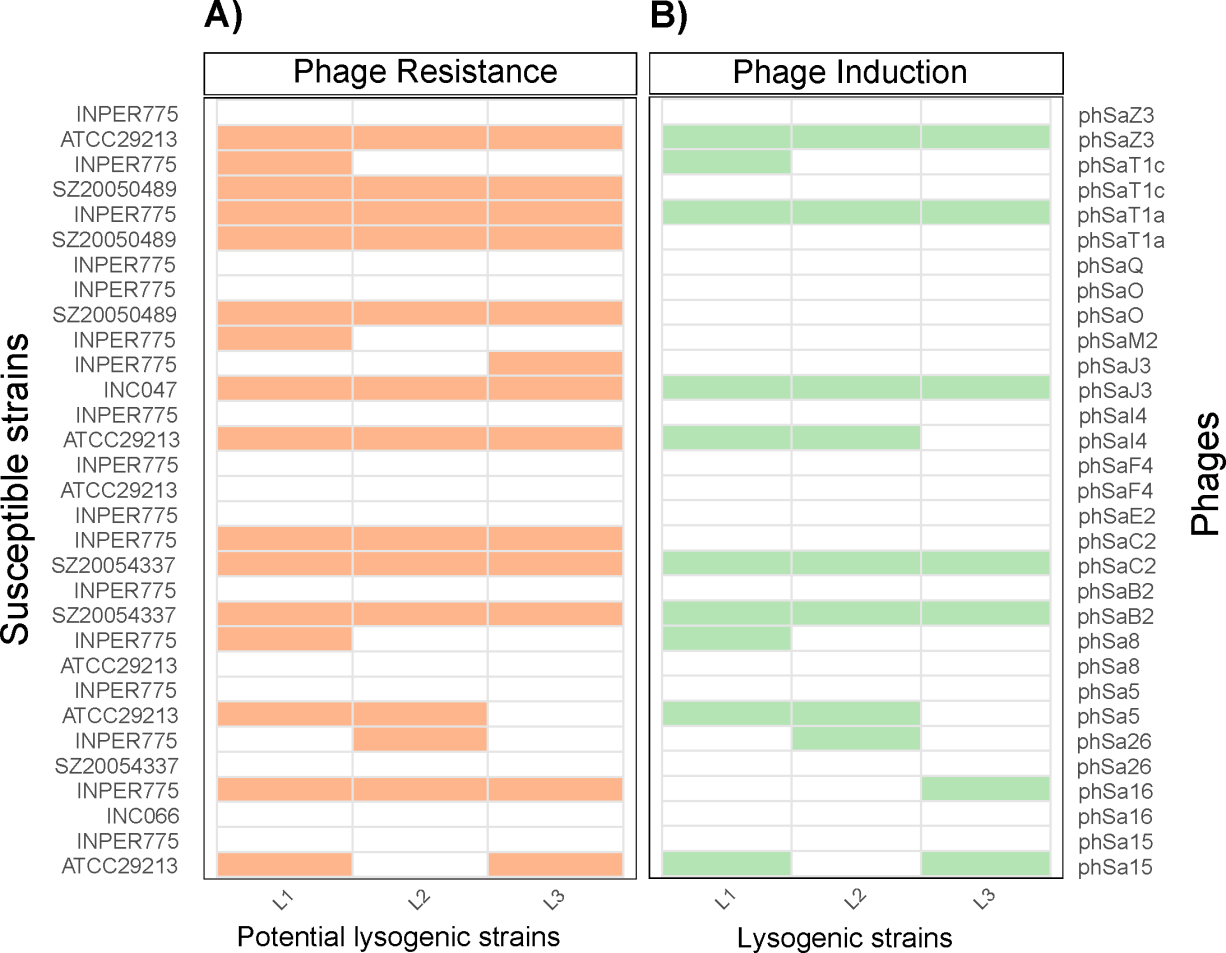
Phenotypes of lysogenic strains obtained experimentally. In panels A and B, the susceptible strains are indicated on the left and the phages on the right. The phenotypes of the three lysogen candidates (L1 to L3, bottom) are indicated at the top of each panel. (**A**) Phage-resistant phenotype: yellow indicates resistance to phage infection, and white indicates susceptibility. (**B**) Prophage induction: green indicates successful MMC-induced prophage induction, and white indicates the absence of prophage induction.

For each phage–host combination, up to three independent colonies (designated L1–L3) were isolated from the lysis zone using spot assays. Susceptibility to the corresponding phages was subsequently assessed via spot assay (Fig. 3A). Of the 31 phage–host combinations analyzed, all three colonies demonstrated resistance to the parental phage in 11 instances. In seven cases, resistance was detected in only one or two of the evaluated colonies. No resistant colonies were identified following phage exposure in the remaining 13 combinations.

To determine whether phage resistance was due to stable lysogeny rather than alternative mechanisms (e.g., spontaneous mutation or receptor loss), all putatively resistant colonies (*n* = 42) were treated with MMC. Phage induction was observed in 60% of these colonies (25/42), as evidenced by the lytic effect of the filtered supernatant on the non-lysogenic host strain in spot-lawn assays (Fig. 3B). These induced phages were subsequently tested against the corresponding putative lysogenic strains, which demonstrated resistance to phage infection, consistent with stable lysogeny (see Fig. S5C at https://github.com/aguayo-alan/Aguayo-Gonzalez_et_al_mSphere_2026). Overall, 12 of the 17 phages demonstrated the capacity to infect and integrate into new host genomes, establish a stable lysogenic state, reenter the lytic cycle, and infect other strains.

To demonstrate that the phage is temperate, whole-genome sequencing was performed on all 12 putative lysogens to assess prophage integration. The genomes were assembled using Unicycler v0.5.1 (27). Chromosomal prophage insertion was confirmed in nine of the 12 evaluated phage–host pairs through targeted searches for phage-derived contigs. There was complete synteny between the chromosomal contigs containing the integrated prophage and the corresponding phage genome sequence of the purified virion (see Fig S6 at https://github.com/aguayo-alan/Aguayo-Gonzalez_et_al_mSphere_2026). In the 12 cases, the boundaries of the prophage were established by identifying the *att* sites with PHASTER (see Table S3 at https://github.com/aguayo-alan/Aguayo-Gonzalez_et_al_mSphere_2026). For the remaining three prophages, fragmented and no complete contigs were recovered, probably because of insufficient sequencing depth and genomic coverage. However, it cannot be ruled out that the phage was not integrated due to the activity of antidefense systems.

Additionally, in some cases, resistance was observed without evidence of phage induction, suggesting that alternative resistance mechanisms, such as mutations or anti-phage defense mechanisms, may underlie the observed phenotypes. No resistant or lysogenic strains were obtained for phSaE2, phSaF4, phSaM2, phSaO, or phSaQ (Fig. 3A through B).

### Core lysogeny genes in induced prophages

To understand the genetic basis of lysogeny in *S. aureus*, the genomic sequences of 17 induced phages were obtained, and the predicted Open Reading Frames (ORFs) were annotated using Pharokka v1.4.0 (28). The genomes had an average size of 44.02 ± 1.53 kb (42.38–45.89 kb) and an average GC content of 34.48% (33.27–35.59%) (see Table S3 at https://github.com/aguayo-alan/Aguayo-Gonzalez_et_al_mSphere_2026). Across the 17 phage genomes, 1,290 (ORFs) were identified, with individual genomes encoding between 73 and 81 ORFs (average: 76 ORFs per genome) (see Table S4 at https://github.com/aguayo-alan/Aguayo-Gonzalez_et_al_mSphere_2026). A high proportion of the predicted ORFs (46%) were hypothetical proteins of unknown functions. Remarkably, 70 ORFs were annotated as transcriptional regulators, indicating a rich repertoire of regulatory elements that may be involved in maintaining lysogeny or controlling the switch to the lytic cycle. Among them, 19 transcriptional repressors and 16 anti-repressors (including seven annotated as anti-repressor Ant) were identified, further supporting the presence of systems that modulate prophage activation. The core elements of the integration-excision machinery were also prevalent, with 17 integrases and six excisionases detected across the genomes. Specialized repressors, such as Arc-like (*n* = 2) and CI-like repressors (*n* = 1), were present (see Table S4 at https://github.com/aguayo-alan/Aguayo-Gonzalez_et_al_mSphere_2026), suggesting the conservation of regulatory modules, such as those found in classical temperate phages. Supporting their integrative capacity, *att* sites, a hallmark of site-specific recombination, were identified in 16 of the 17 phages, further confirming their temperate nature (see Fig. S3 and Table S3 at https://github.com/aguayo-alan/Aguayo-Gonzalez_et_al_mSphere_2026).

### Genomic classification of prophages, temperate phages, and SAPIs

**Fig 4:**
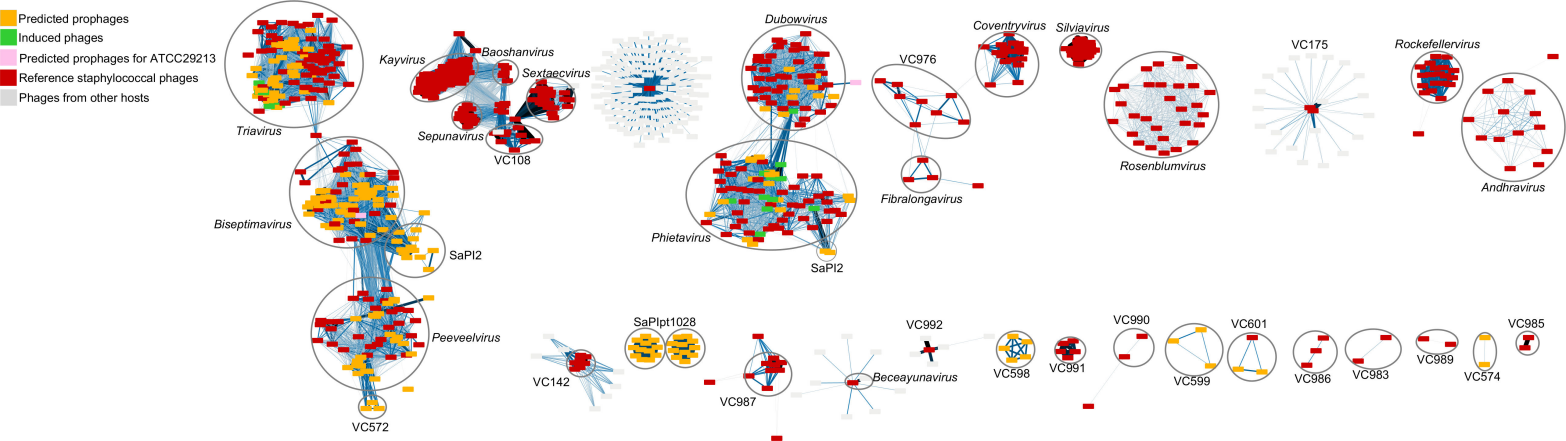
Viral clusters (VCs) of *S. aureus* phages and prophages. Cytoscape network representation of viral clusters (VCs) based on shared protein family content, as determined by vConTACT2. Each node represents a phage or prophage genome, and the edges indicate the similarities in protein content. Known VCs were identified using the names of the VCs and genus name from the ICTV, when available. VCs identified as SaPISs are encircled. Singletons were excluded from the network. The predicted prophages from 109 clinical *S. aureus* genomes are shown as orange squares, and the 17 inducible prophages isolated in this study are highlighted in green. Reference phages retrieved from the NCBI Viral Genome Database are shown in red (inset).

To contextualize the prophages and phages within established classifications in existing databases, all viral elements were classified using vConTACT2, which clusters phages based on shared protein family content as a proxy for their overall genomic relatedness (23). Using this approach, 789 viral elements, including 556 reference phages, 175 predicted prophages, 41 predicted SaPIs, and 17 inducible phages, were resolved into 38 viral clusters (VCs) (Fig. 4; also see Fig. S7 at https://github.com/aguayo-alan/Aguayo-Gonzalez_et_al_mSphere_2026). These VCs represent network-defined units of viral diversity and correspond to established taxonomic groupings (24, 29).

The experimentally induced temperate phages recovered in this study were predominantly affiliated with the genera *Triavirus*, *Phietavirus*, and *Dubowvirus* (Fig. 4), indicating that inducible, infection-competent phages circulating in local *S. aureus* populations largely belong to these well-characterized genera. In contrast, *Biseptimavirus* and *Peeveelvirus* were represented exclusively by predicted prophages that were absent among experimentally induced phages, suggesting reduced inducibility under MMC treatment or lineage- and context-specific regulatory constraints that limit prophage excision and replication.

Within the genus *Phietavirus*, prophages segregate into two distinct genomic clusters: the first cluster comprised 45 members distributed across four subclusters (VC571.0 to VC571.3), including nine inducible phages (phSaF4, phSa5, phSa8, phSa15, phSaO, phSaI4, phSaT1a, phSaT1c, and phSaC2). These phages displayed high genomic synteny with the reference phage *Phietavirus* pv3MRA, particularly across structural gene modules, terminase complexes, and lysis-associated regions (see Fig. S8C at https://github.com/aguayo-alan/Aguayo-Gonzalez_et_al_mSphere_2026). The second lineage encompassed three subclusters (VC569.0 to VC569.2) comprising 24 members, including the inducible phage phSa26, which showed strong similarity to *Phietavirus* ETA, StauST3985, SAP27, and pv80, particularly within the structural and DNA-processing modules (see Fig. S8D at https://github.com/aguayo-alan/Aguayo-Gonzalez_et_al_mSphere_2026). The marked divergence between these two lineages suggests the presence of distinct evolutionary trajectories within *Phietavirus*, potentially warranting future taxonomic refinement.

Notably, none of the 17 experimentally inducible phages recovered in this study were affiliated with *Biseptimavirus* or *Peeveelvirus*, despite the widespread presence of these genera in the *in-silico* analysis. *Biseptimavirus* was distributed across four subclusters in the VConTACT2 network (74 members), whereas *Peeveelvirus* formed a single cluster comprising 39 members. This discrepancy may reflect the constraints of the experimental induction approach, which relied on a single stressor (MMC) or lineage- and context-specific regulatory mechanisms not captured under the conditions tested here.

Among the viral clusters predicted through vConTACT2 analysis of local *S. aureus*, we identified two clusters, VC600 and VC570, which exhibited no detectable similarities to previously characterized *Staphylococcus* phage sequences. As described above, they represent MGES that exploit phage-derived machinery that we identified as SaPIs by their integrase genes (see Table S5 at https://github.com/aguayo-alan/Aguayo-Gonzalez_et_al_mSphere_2026). Furthermore, this analysis revealed that 41 of the predicted prophage regions encoded integrases corresponding to the known SaPIpt1028, SaPI1, and SaPI2 (see Table S6 at https://github.com/aguayo-alan/Aguayo-Gonzalez_et_al_mSphere_2026).

Integrases specific to SaPIpt1028 were identified in 24 elements, including representatives from both VC570 and VC600, as well as a singleton prophage not assigned to any cluster in the vConTACT2 network (Fig. 4). SaPI1 was identified as a single element (INPER691_001_f6_m), whereas SaPI2-related integrases were detected in 16 predicted elements, spanning 15 elements distributed across five viral clusters (VC1.3, VC1.4, VC2, VC3, VC569) and one singleton.

In the global collection, 222 SaPI elements (see Table S7 at https://github.com/aguayo-alan/Aguayo-Gonzalez_et_al_mSphere_2026) represented five distinct SaPI types: SaPI2 (*n* = 106), SaPIpt1028 (*n* = 77), SaPI1 (*n* = 16), SaPImw2 (*n* = 12), and SaPIbov1 (*n* = 11). These findings indicate that SaPI elements are frequently misclassified as prophages in large-scale genomic surveys and underscore the need for targeted screening to accurately distinguish between phage genomes and phage-related pathogenicity islands in *S. aureus*.

### Virulence factors in prophages and SaPIs

**Fig 5:**
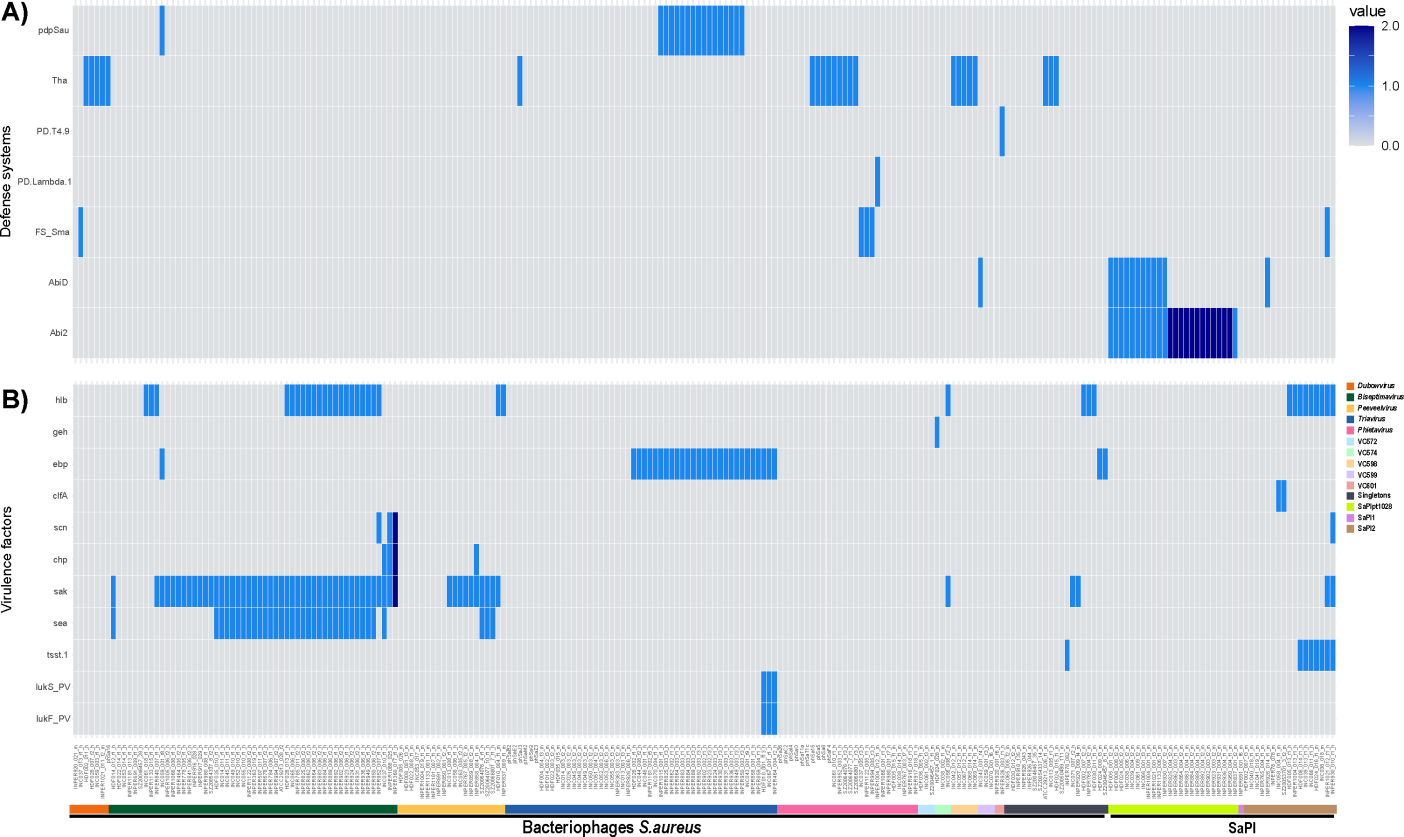
Distribution of anti-phage defense systems and virulence factors in *S. aureus* phages, prophages, and SaPIs. Heatmaps showing the presence of defense systems (**A**) and virulence factors (**B**) in phages, prophages, and staphylococcal pathogenicity islands (SaPIs). Both heatmaps share the same *x*-axis labels at the bottom, identifying individual phages, prophages, and SaPIs, which are grouped by taxonomic classification (indicated by the colored headers). The *y*-axis labels on the left indicate the specific anti-phage defense systems (**A**) or virulence factors (**B**). The color scale represents the copy number, from white, which indicates absence, to dark blue, which indicates two copies.

To assess the virulence potential of the prophages, viral clusters (VCs) were screened for virulence-associated genes using ABRicate (see Materials and Methods). Inducible prophages affiliated with the genera *Triavirus*, *Phietavirus*, and *Dubowvirus* generally lacked identifiable virulence factors, except for a subset of *Triavirus* members (Fig. 5). Consistently, phages assigned to *Phietavirus* and *Dubowvirus* in public databases also lack virulence-associated genes, suggesting functional conservation within these taxa.

In contrast, several *S. aureus* strains associated with VCs classified as *Triavirus*, *Biseptimavirus*, and *Peeveelvirus* harbored prophage-linked virulence determinants. *Triavirus* prophages encode LukS/LukF subunits of Panton–Valentine leukocidin, whereas members of the *Peeveelvirus* genus carry genes encoding staphylokinase (Sak) and enterotoxin A (Sea). *Biseptimavirus* prophages displayed particularly diverse virulence repertoires, including immune evasion factors such as Sak, staphylococcal complement inhibitor (Scn), chemotaxis inhibitory protein (Chp), and Sea toxins (Fig. 5).

In this cohort, SaPI types showed distinct gene associations, as inferred from draft regions and integrase-based typing (Fig. 5). Some SaPI2 elements harbor TSS-1 (toxic shock syndrome toxin), which are recognized as being carried by SaPIs and other pathogenicity islands (30, 31). In contrast, islands identified as SaPIpt1028 lacked associated virulence genes, which agrees with previous reports indicating that these islands preferentially carry anti-phage gene systems (Sma and Abi systems) rather than virulence factors (32) (see Table S6 at https://github.com/aguayo-alan/Aguayo-Gonzalez_et_al_mSphere_2026). Here, we detected a variant of SaPIpt1028 mainly associated with the Abi2/AbiD systems in two clonal complexes, whereas the Sma system was integrated into other prophages (Fig. 5).

### Phage/SaPI distribution in relation to host phylogeny

**Fig 6:**
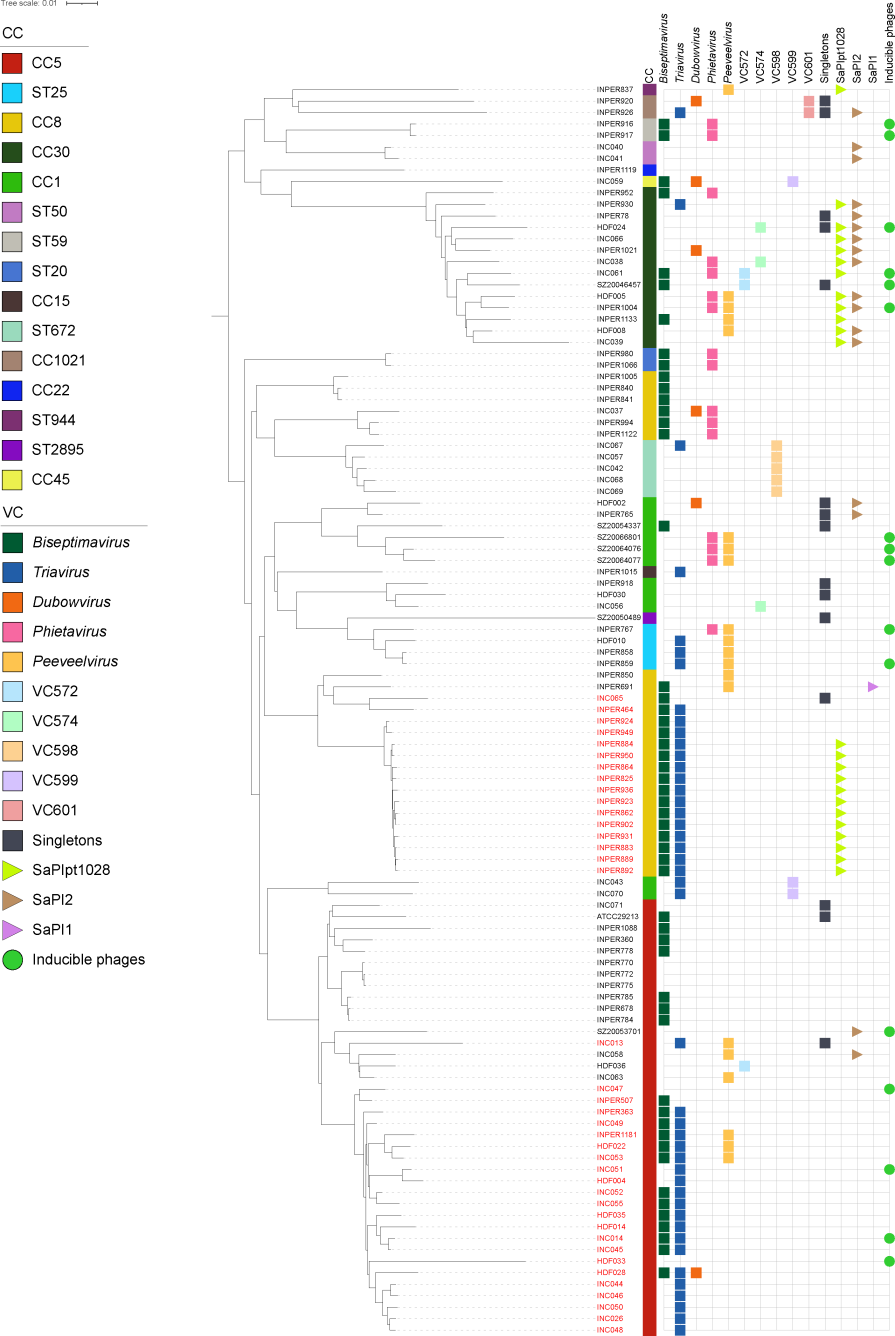
Phylogenetic relationships and prophage integration patterns in the host strains. A phylogenetic tree of 109 local *S. aureus* genomes. The tree displays isolate names (black for MSSA and red for MRSA) at the tips, with clonal complexes (CC) indicated for each strain. Prophage groups are represented by colored squares corresponding to the predicted prophages found in each strain, including the viral clusters (VC) identified in the data set. SaPIs are shown as colored triangles, and inducible phages are represented by circles. Prophages and SaPIs were mapped adjacent to their respective host strains in the tree. The color keys for all elements are provided in the figure legends.

To understand the relationship between host strains and the presence of different prophage groups as well as SaPIs, a maximum likelihood phylogenetic tree was constructed using the core proteins (*n* = 2,108) obtained from the pangenome model inferred with BPGA v1.3 (see Materials and Methods) (33), using genomes from 109 local strains. The strains were grouped into clades corresponding to their clonal complexes, with CC5, CC8, and CC30 being the most common. When mapping the various phage groups onto the phylogeny, the prophages belonging to the *Peeveelvirus, Biseptimavirus*, and *Triavirus* were widespread across five to eight different clades with both MRSA and MSSA strains. In contrast, *Dubowvirus* and *Pietavirus* were present only in CCs, which grouped MSSA strains, although in different clades. Other less-represented VCs, such as VC57, VC574, VC589, and VC601, were associated with specific clades in the phylogeny, suggesting a restricted host range. Consistent with the data in Fig. 6, MSSA strains contained a more diverse set of prophages. Along with prophage distribution, the pathogenicity island SaPIpt1028 was detected in both methicillin-resistant CC8 and CC30 MSSA isolates, whereas SaPI2 was only detected in CC30 in this data set (Fig. 6).

Altogether, these results indicate a rich mobilome landscape that deserves further studies of their interactions and functional levels.

## DISCUSSION

This study demonstrated that only a relatively small fraction of the prophages predicted in *S. aureus* genomes was functionally active by MMC induction. Although nearly all *S. aureus* strains analyzed carried between one and four prophages, as predicted by the VIBRANT tool, only a limited subset (17 of 109 isolates) produced inducible phages following MMC treatment. Remarkably, the induced phages lysogenize new *S. aureus* hosts and are reinduced thereafter, demonstrating that they remain fully functional and transmissible.

Two main explanations may account for the observed low inducibility pattern. First, many prophages may require alternative inducers—chemical, environmental, or stress-related—that differ from MMC and are SOS independent (15). For instance, phage ϕphiMBL3 of *S. aureus* ATCC 6341 has been shown to respond specifically to pyocyanin, a natural compound produced by *Pseudomonas aeruginosa* (14). It directly triggers the oxidative stress response, which converges in the SOS response to activate the prophage. However, MMC exposure alone cannot induce this prophage (34). Other prophages respond to quorum sensing and more complex growth conditions, such as growth in bile acids, to reveal their activity (16, 35). Second, some prophages may become defective through the loss or mutation of key regulatory or structural genes necessary for excision, replication, and particle formation. Nonetheless, prior evolutionary analyses suggest that prophage genes remain under purifying selection at rates comparable to those of housekeeping genes, implying a retained functional potential that might only manifest under specific conditions (36). These observations suggest the need for a broader search for inducers to complete the functional mapping of prophages in polylysogenic *S. aureus* (15).

In our study, although *S. aureus* strains frequently carried multiple prophages, typically only one was induced under the experimental conditions. Polylysogeny raises questions regarding the selective induction of a single prophage. It is likely that the winning prophage outcompetes other resident prophages through different molecular mechanisms and growth conditions (15). Within intracellular environments, interactions among prophages and with other mobile genetic elements (such as SaPIs) may, in some instances, inhibit the full expression of early genes (37). Variations in the alleles encoding phage proteins, including repressors and antirepressors, can influence the efficiency of phage production. At the translational level, competition for metabolites necessary for phage synthesis may also restrict the predominance of a prophage in the production of viral particles (15).

The prophages identified in this study did not contain detectable virulence genes, indicating a limited influence on *S. aureus* pathogenicity. This observation is consistent with previous findings, which show that most *S. aureus* prophages available in public databases, such as GenBank and INPHARED, are temperate and lack prominent virulence determinants, as assessed by BACPHLIP (38, 39). Nevertheless, these results differ from those of earlier studies highlighting the role of phages in pathogenicity evolution through the carriage of diverse toxins and immune evasion factors (40). Additionally, they contrast with bioinformatic analyses of viromes and metagenomes, which frequently report the presence of antibiotic resistance and virulence genes within the phage sequences (11, 38).

While our study identified predicted prophages associated with virulence genes, we also noted instances in which virulence genes were potentially misassigned to prophage regions. For instance, *in silico* analyses initially attributed the *hlb* gene, which encodes β-hemolysin, to the prophage genome; however, subsequent sequence alignment revealed that *hlb* is situated adjacent to the integrase gene at the canonical *attB* site, indicating host gene disruption rather than authentic prophage incorporation (see Fig. S9A at https://github.com/aguayo-alan/Aguayo-Gonzalez_et_al_mSphere_2026). Similarly, the *ebp* toxin gene was originally predicted within a prophage region, but further boundary analysis demonstrated that the *ebp* locus is located several open reading frames downstream of the lysis module and lies outside the canonical phage genome. These findings highlight the inherent limitations of existing prophage prediction algorithms, particularly their difficulty in accurately defining prophage boundaries and their tendency to incorporate adjoining chromosomal regions (see Fig. S9B at https://github.com/aguayo-alan/Aguayo-Gonzalez_et_al_mSphere_2026).

The inducible prophages belong to the phage genera *Triavirus*, *Phietavirus*, and *Dubowvirus*, which are generally associated with a low incidence of virulence factor carriage in their bacterial hosts. This contrasts with the genus *Biseptimavirus*, which includes phages that encode immune evasion genes and toxins. Although Panton–Valentine leukocidin (PVL) genes (*lukS-PV* and *lukF-PV*) have been found in some *Triavirus* phages, *Phietavirus* members are not currently known to harbor these virulence genes. These patterns reinforce the idea that not all prophages contribute directly to virulence but may play more subtle roles in shaping the ecology and evolution of *S. aureus*.

Our findings also identify the limitations of bioinformatic prophage prediction, particularly in the draft genome assemblies. For example, a functionally inducible phage was recovered from strain HDF033 despite the lack of high- or medium-quality prophage predictions, likely due to low-quality genome assembly. Prophage sequence splitting across contigs can hinder accurate prediction and classification, emphasizing the importance of using high-quality complete genome assemblies for prophage analysis.

An additional layer of complexity arises from the presence of SaPIs, which share structural and functional features with phages and can be mistaken for prophages when using standard prediction tools (11). The detection of elements that were initially predicted as prophages but later identified as SaPIs underscores the intricacy of the *S. aureus* mobilome and the need for careful annotation and complementary approaches, such as experimental induction or comparative genomics, to distinguish true prophages from phage-related elements in the future.

Together, our results revealed that only a small proportion of the prophages predicted in *S. aureus* genomes were functionally active after MMC treatment. Although most inducible prophages do not carry virulence genes, their persistence and functionality suggest broader roles in host adaptation, competition, and genetic exchanges. These findings underscore the importance of combining bioinformatics and experimental approaches to fully understand the dynamics of prophages in clinically relevant *S. aureus* populations and explore the potential of alternative inducers beyond classical DNA-damaging agents.

## MATERIALS AND METHODS

### *S. aureus* isolates and global genome data sets

*S. aureus* isolates were obtained from the Faculty of Medicine of the National Autonomous University of Mexico (FM-UNAM) and the National Institute of Medical Sciences and Nutrition Salvador Zubirán (INCMNSZ). *S. aureus* isolates (101) from FM-UNAM were previously reported and the complete genomes were retrieved from GenBank (see Table S2 at https://github.com/aguayo-alan/Aguayo-Gonzalez_et_al_mSphere_2026). The genomes of *S. aureus* isolates from INCMNSZ were *de novo* sequenced (see below).

For the global prophage diversity analysis, 993 complete *S. aureus* genomes were retrieved from the NCBI GenBank database as of April 2022. Genomes were selected based on assembly quality criteria, including complete genomes, to ensure high-quality sequence data for accurate prophage identification and characterization. Detailed information on the selected genomes is presented in Table S1 at https://github.com/aguayo-alan/Aguayo-Gonzalez_et_al_mSphere_2026. MRSA and MSSA classifications were performed using the SCCmec (41) typing tool camlhmp version 1.1.0 (42), with SCCmec_targets schema version 1.2.0 and sccmec_regions schema version 1.2.0.

### Growth conditions, prophage induction, and host range analysis

*S. aureus* strains were grown in Lysogenic Broth (LB) medium supplemented with 7 mM CaCl₂ and 10 mM MgSO₄ at 37°C with constant shaking at 210 rpm. Bacto agar was added to the medium when solid plates were needed.

Phages were induced by treatment with MMC (0.5 μg/mL) during the early logarithmic phase of the culture. After 8 h of incubation, the cultures were centrifuged at 3,000 × *g* for 10 min, and the supernatant was collected. To remove cell debris, chloroform was added to the supernatant at 10% (vol/vol), followed by centrifugation under the same conditions. The resulting supernatant was filtered through a 0.22 μm membrane filter. The final supernatant was collected and stored at 4°C until further use. Phage detection was performed using a double-layer plaque assay technique with the collected strains. A total of 200 μL of each sample and 100 μL of overnight culture were added to 4 mL of top agar (soft medium with 0.4% agar melted at 42°C) and poured onto Luria-Bertani (LB) plates. The appearance of lytic plaques was visually evaluated, and isolated plaques were recovered and purified in three replicates.

For host range analysis, a panel of 40 *S. aureus* strains was assembled to identify permissive hosts capable of supporting phage propagation. Because we did not have access to a universal *S. aureus* indicator strain, host range determination was performed using an indirect spot test approach. Prior to inclusion in the host range panel, all strains were independently screened for lysis after undergoing MMC treatment. Strains were selected primarily based on the lack of detectable viral particle production under induction conditions and the absence of bioinformatically predicted prophages.

Spot tests were performed by depositing aliquots of the induced, filtered supernatants onto bacterial lawns prepared with each strain in the panel. The plates were incubated at 37°C and examined for zones of lysis indicative of phage infection.

### Genomic sequencing of bacteria and bacteriophages

In this study, we sequenced the genomes of eight *S*. *aureus* isolates from the INCMNSZ and 17 temperate phages. Bacterial DNA was obtained using the GenElute Bacterial Genomic DNA Kit (Sigma Aldrich) following standard protocols, with lysostaphin treatment (200 units/mL) for cell lysis and RNase A for RNA removal. DNA quality and concentration were assessed using agarose gel electrophoresis, spectrophotometry (NanoDrop 2000; Thermo Fisher, Waltham, MA, USA), and fluorometry (Qubit dsDNA assay; Thermo Fisher). Whole-genome sequencing was performed at BGI Tech Solution using DNBSEQ technology with paired-end 150 bp reads from 350 bp insert libraries, generating 2 Gb per sample. Raw sequencing data underwent quality control and trimming using FastQC v0.11.8 and Trim Galore v0.6.4, followed by *de novo* assembly using the SPAdes genome assembler v3.13.1 (43).

Phage DNA was purified following the protocol of a DNA isolation kit for cells and tissues (Roche Life Sciences, Basel, Switzerland) with modifications. The raw sequencing data were subjected to quality control using FastQC v0.11.9 and cleaned using Trim Galore v0.6.7. *De novo* assembly was performed using SPAdes Genome Assembler 3.13.1 (43), MEGAHIT v1.2.9 (44), and Unicycler v0.5.1 (27). Assembly quality metrics were evaluated using QUAST v5.3.0 (45), and the best assembly for each phage was selected based on these metrics. Subsequently, genome completeness was assessed using CheckV v.1.0.1 (46).

### Prophage prediction

The prophage content prediction in *S. aureus* genomes was conducted using the bioinformatics tool Virus Identification By iteRative ANnoTation (VIBRANT v1.2.1 (22). Default parameters were applied, considering prophage sequences with a minimum length of 1,000 bp and an open reading frame (ORF) count of four or more ORFs.

Prophage counts in MSSA and MRSA isolates were compared using generalized linear mixed-effects models (GLMMs) with a Poisson error distribution, implemented in R v4.5.1 using the glmmTMB v1.1.13 package (47). To adjust for phylogenetic relatedness among the bacterial strains, clonal complex assignments were used as random intercepts in the model. MSSA isolates were the reference category for all statistical comparisons, with significance set at α = 0.05. Data were visualized as boxplots using ggplot2 v4.0.1, with statistical summaries (β estimates, standard errors, and *P*-values) directly annotated on the figure.

### Genome annotation and viral cluster construction

The prediction and annotation of open reading frames (ORFs) for the isolated phages and predicted prophages were performed using Pharokka v1.4.0 (28), followed by manual curation of the results. Attachment sites were identified using the PHASTER tool (48).

Viral clusters (VCs) were generated using vConTACT v.2 (23, 24) to construct the phage similarity network. vConTACT2 groups similar proteins into protein clusters (PCs) using the Markov Clustering Algorithm (MCL) and calculates the maximum likelihood of shared PCs (edges) between genomes (nodes), resulting in a bipartite network. VCs were identified using ClusterONE with a significance threshold of 60. The resulting network was visualized using Cytoscape v.3.10.2 (49).

### Experimental lysogeny

Four host strains (INPER775, ATCC 29213, SZ20054337, and INC047) were selected for lysogeny screening based on their host-range profiling. INPER775 was included because of the absence of predicted prophage regions, whereas ATCC 29213 and SZ20054337 were incorporated after failing to produce inducible prophages following MMC treatment. INC047 was selected because of its high susceptibility to phSaJ3. These characteristics, along with their susceptibility profiles, guided the selection of 31 phage–host combinations for downstream lysogeny evaluation. Combinations were chosen based on their lytic activity during liquid culture growth and the formation of distinct lysis zones in spot assays, but turbidity (resistant cells) began to appear after repeated passages or extended incubation times.

For lysogen isolation, 10 μL of each phage lysate was spotted onto lawns of the corresponding sensitive host strain and incubated for 12 h at 37°C. Viable cells from the central region of the lysis zones were recovered using sterile bacteriological loops and streaked for isolation on a solid medium. Three independent colonies (L1–L3) were collected for each candidate combination and purified through three sequential passages for further analysis.

Purified isolates were grown in broth and evaluated for superinfection immunity by spot testing with the parental phage, confirming resistance to reinfection. Isolates exhibiting immunity were subjected to prophage induction assays using MMC (0.5 μg/mL, 8 h) to determine their capacity to enter the lytic cycle.

Prophage insertion was verified by targeted searches for phage-derived sequences within the assembled bacterial genomes. Integration events were further validated by synteny analysis between each lysogenic strain and its corresponding phage genome using EasyFig v2.2.5 (50), which enabled the visualization of conserved genomic architecture and insertion boundaries.

### Identification of SaPI integrases

Pathogenic islands (SaPIS) were searched for in the high- and medium-quality prophage predictions obtained using VIBRANT. The complete genomic sequences of all predicted prophages were queried using BLASTp against a curated database of SaPI integrase genes (Table S5). BLASTp parameters were set to a minimum of 90% amino acid identity and 80% alignment coverage. The predicted prophages that met these criteria were redefined as SaPIs.

### Phylogenetic analysis and mobile element mapping

Phylogenetic analysis of 109 local *S. aureus* strains was performed using BPGA v1.3 (33) with the core genome approach, identifying 2,108 core proteins through a comparative genomic analysis. Maximum likelihood phylogenetic reconstruction was conducted using IQ-TREE v2.1.2 with default parameters, implementing the JTT + F + R3 evolutionary model and 1,000 bootstrap replicates to assess branch support.

Multilocus sequence typing (MLST) was used for clonal complex (CC) assignment. Sequence types (STs) were primarily determined using the BV-BRC Genomic Annotation Service by comparing them with established ST alleles in PubMLST (https://pubmlst.org/). For novel alleles absent from PubMLST, the Center for Genomic Epidemiology MLST service (https://cge.food.dtu.dk/services/MLST/) was used with default parameters and BV-BRC-derived FASTA files to determine the allelic profiles and corresponding STs. Related STs were subsequently grouped into CCs using the eBURST algorithm (https://pubmlst.org/organisms/staphylococcus-aureus), which clusters strains that share similar allelic profiles.

The resulting phylogenetic trees were drawn and edited using the Interactive Tree of Life program (iTOL v7.2). Prophage groups, SaPIs, and other mobile elements were mapped onto a phylogenetic tree using the iTOL annotation features.

### Identification of virulence factors, antibiotic resistance genes, and anti-phage systems

Identification of virulence factors and antibiotic resistance genes was carried out using ABRicate 1.0.1 (51) with default parameters (80% identity and 80% coverage), utilizing the Virulence Factor Database (VFDB) (52) and Comprehensive Antibiotic Resistance Database (CARD) (53), respectively. The search for anti-phage defense systems was performed using DefenseFinder 1.3.0 (54) with default parameters.

## Data Availability

All relevant data supporting the findings of this study are available in the main text and the supplemental files available at https://github.com/aguayo-alan/Aguayo-Gonzalez_et_al_mSphere_2026. Genomic sequences from bacterial strains and phages are publicly available through the National Center for Biotechnology Information (NCBI), with accession numbers detailed in the supplemental material.
